# Trace phenol-formaldehyde resin activation mechanism of intermediate graphitic layer removal in carbon for enhanced Li-ion capacitor performance

**DOI:** 10.3389/fchem.2025.1592695

**Published:** 2025-09-22

**Authors:** Yingkai Xia, Shuang Wei, Xiao Wei, Yuehui Chen, Jiahang Ding, Haoyuan Zheng, Sen Yang, Shaobin Yang

**Affiliations:** 1 School of Mining, Liaoning Technical University, Fuxin, Liaoning, China; 2 College of Material Science and Engineering, Liaoning Technical University, Fuxin, China; 3 College of Science, Liaoning Technical University, Fuxin, Liaoning, China

**Keywords:** porous carbon electrode material, activated extraction layer, heat treatment, microporous and mesoporous materials, li-ion capacitor

## Abstract

Precise modulation of the pore structure in activated carbon can further enhance the capacitance performance of supercapacitors. As a carbonaceous precursor, phenol-formaldehyde resin (PR) plays a dual role in both carbon deposition and activation for pore regulation; however, the activation mechanism governing its pore-tuning effect remains unclear. In this study, trace PR with a mass ratio of 0.2%–0.8% was mixed with activated carbon for heat treatment. The results revealed that trace amounts of PR exhibit an activation mechanism by selectively removing intermediate graphene layers. Specifically, the removal of one–three graphene layers resulted in the formation of periodic micropores with diameters of 0.50–0.56 nm, 0.81–0.90 nm, and 1.14–1.19 nm. Correlation analysis demonstrated that the pore size most strongly associated with lithium-ion capacitance and diffusion coefficients fell within the range formed by the removal of a single graphene layer. Compared with one-step activation using PR, the multi-step activation process slowed the rate of pore expansion following single-layer removal, facilitating the formation of a greater proportion of 0.54 nm pores—those most closely linked to enhanced capacitance and ion diffusion. Consequently, the prepared coal-derived activated carbon achieved a capacitance of 164 F g^-1^, matching the highest reported values for aqueous lithium-ion capacitors using porous carbon (PC) materials. This study reveals a novel mechanism of precise pore modulation at the 0.01 nm scale through trace PR activation, providing new insights into the structural regulation of PC materials for advanced energy storage applications.

## Graphical Abstract

**Figure:**
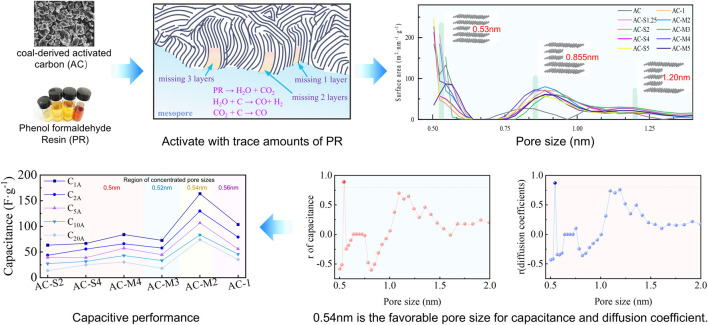

## 1 Introduction

In recent years, Li-ion capacitors (LICs) have garnered significant attention in the field of supercapacitors due to their remarkable high power density compared to lithium-ion batteries (LIBs) (Jiang et al., 2022; Soltani and Beheshti, 2021). However, a major challenge lies in their relatively lower energy density. Capacitor electrode materials primarily consist of carbon-based materials (Liang and Wang, 2022; Wang et al., 2019) that store energy via electric double-layer capacitance, and non-carbon materials (Khandare et al., 2023; Khandare et al., 2024; Ramachandran et al., 2022; Ramachandran et al., 2023a; Ramachandran et al., 2023b) that store energy through pseudocapacitance mechanisms. Currently, PC widely regarded as a promising electrode material for supercapacitors, can be derived from various carbonaceous sources, including coal (Dong and Xiao, 2023; Ghosh and Lee, 2012), pitch (Song et al., 2018), biomass materials such as wood (Bi et al., 2019) and coconut shells (Sesuk et al., 2019), as well as organic polymers (Cho et al., 2022; Zhang et al., 2018). Among these, coal stands out as a particularly attractive precursor for the preparation of PC for LICs due to its abundant reserves and cost-effectiveness.

PC used for capacitor electrodes is typically synthesized via one-step chemical activation methods using agents such as KOH (Tseng and Tseng, 2005), ZnCl_2_ (Li et al., 2020), and CO_2_ (Guo et al., 2009). However, the resulting pore structures are often irregular and generally require further fine-tuning. A commonly employed mechanism for pore adjustment is carbon deposition, where carbon precursors such as phenol-formaldehyde resin, phenol, or tar are deposited to modulate the pore structure (Dryfe, 2006; Paredes et al., 2021). This approach typically leads to a reduction in pore size and specific surface area (Paredes et al., 2021), consequently resulting in decreased pore volume, which can negatively impact capacitive performance. Another strategy for pore structure refinement is carbon activation, which involves high-temperature treatment with activating agents such as H_2_O, CO_2_, ionic salts, or phenol-formaldehyde resin (Guo et al., 2024; Kyotani, 2000). This method promotes the formation of new micropores, leading to increased specific surface area and pore volume, thereby enhancing capacitance. Notably, phenol-formaldehyde resin can function as both a carbon deposition agent (Tennison, 1998) and an activation agent for pore regulation. While considerable research has been dedicated to the carbon deposition mechanism, limited studies have investigated the combined effects of activation and pore regulation on LIC performance.

In the study of PC materials for supercapacitor electrodes, Gogotsi (Chmiola et al., 2006), Huang (Huang et al., 2008), and others have demonstrated that when the pore size is below 1 nm, smaller pores generally lead to higher capacitance. On the other hand, recent research has shown that in ultra-micropores smaller than approximately 0.7 nm, reduced pore size significantly increases ion diffusion resistance, thereby deteriorating the power performance of the supercapacitor (Dvoyashkin et al., 2021; Jian et al., 2022). Therefore, achieving precise control over carbon micropore structures is critically important for enhancing their capacitive performance.

In this study, coal-derived activated carbon was utilised as the precursor, and a series of multi-step thermal treatments with trace amounts of PR were conducted to examine the evolution of pore structure and carbon layer morphology. By correlating pore structural characteristics with carbon layer configuration, we elucidated a graphitic layer removal activation mechanism. Furthermore, a systematic analysis of the relationship between carbon electrode capacitance and pore architecture enabled the identification of the optimal pore structures for facilitating Li-ion storage and diffusion. As a result, we successfully developed a coal-derived activated carbon material exhibiting superior Li-ion capacitive performance, surpassing the highest reported values in the literature. This study demonstrates the effectiveness and simplicity of multi-step layer removal activation as a powerful strategy for tailoring pore structures to enhance LIC performance.

## 2 Experimental section

### 2.1 Materials

In this study, lean coal from Shanxi was uniformly mixed with KOH (China National Pharmaceutical Group, Beijing, China) at a mass ratio of 1:3. Under a nitrogen atmosphere, the mixture was kept at 800 °C for 2 h, washed, and dried to obtain coal-derived PC, named AC. AC was used as the experimental raw material. PR was sourced from China National Pharmaceutical Group. LiOH came from Shanghai Macklin Biochemical Technology Co., Ltd. ,Shanghai, China. Trace amounts of PR were dissolved in 1 L of deionized water to prepare solution for later use.

### 2.2 Preparation of coal-derived porous carbon

#### 2.2.1 Activation

Lean coal powder, with a particle size under 125 μm, was thoroughly mixed with KOH at a mass ratio of 1:3. The mixture was then heated under a N_2_ atmosphere to 800 °C for 2 h. The resulting product was then treated with 1 M HCl followed by deionized water washing, and ground to <50 μm to obtain sample AC.

#### 2.2.2 Single-step heat treatment

PR was employed as the precursor for carbon deposition. AC was mixed with a designated amount of PR in a 20 mL aqueous solution. After thoroughly mixing, the solution was allowed to stand, filtered, and dried. The resultant sample was heated under N_2_ atmosphere to 800 °C for 2 h and cooled to 25 °C. The cooled sample was then washed, dried, ground and sieved to obtain a carbon deposition sample with a particle size of less than 40 μm. A smaller quantity 0.002 g of PR was used to create the AC-1 sample, whereas a larger quantity 0.008 g was used for the AC-S4 sample. The specific experimental conditions are detailed in Table 1.

**TABLE 1:** Preparation methods of coal-derived porous carbon.

Sample	Raw material	PR (g)	Mass ratio (%)	Temperature (°C)
AC-1	1 g AC	0.002	0.2	800
AC-S1.25	1 g AC	0.0025	0.25	800
AC-S2	1 g AC	0.004	0.4	800
AC-S4	1 g AC	0.008	0.8	800
AC-S5	1 g AC	0.010	1.0	800
AC-M2	1 g AC-1	0.002	0.4	800
AC-M3	1 g AC-M2	0.002	0.6	800
AC-M4	1 g AC-M3	0.002	0.8	800
AC-M5	1 g AC-M4	0.002	1.0	800

#### 2.2.3 Multi-step heat treatment

The mixture containing 1 g of AC-1 and 0.002 g of PR in a 20 mL aqueous solution underwent carbon deposition again, resulting in the AC-M2 sample. This procedure was repeated to produce the AC-M3 and AC-M4 samples. The total amount of PR introduced in both the AC-S4 and AC-M4 samples amounted to 0.008 g. All carbon-deposited samples in this study are classified as coal-derived porous carbon.

### 2.3 Analysis and characterization

The X-ray diffraction analysis of the samples was conducted using a XRD-6100X (Shimadzu, Tokyo, Japan) diffractometer with a Cu target (λ = 0.154184 nm), a scan speed of 10°·min^−1^, a step size of 0.02°, and a diffraction angle range of 5°–80°. Microstructural analysis was performed using scanning electron microscopy (SEM) and transmission electron microscopy (TEM) with a JSM-7500F and a JEM-2100F (JEOL Beijing, China) instrument, respectively. Nitrogen adsorption analysis was conducted at 77 K using an AutosorbiQ (Anton Paar, Graz, Austria). Surface functional group analysis was performed using an IR Prestige-21 (Shimadzu, Tokyo, Japan) with KBr and a 250 (Thermo Fisher Escalab, 100 Technology Drive, Waltham, MA 02451) X-ray photoelectron spectrometer with an Al Ka X-ray source (h = 1486.7 eV).

### 2.4 Electrochemical performance testing

A slurry was prepared by combining coal-derived PC, a conductive agent (acetylene black), and a binder (polytetrafluoroethylene, PTFE) in a mass ratio of 8:1:1. This mixture was then uniformly coated onto a 1 cm × 1 cm piece of Ni foam, dried at 60 °C for 12 h, and subsequently compacted under a pressure of 4 MPa to fabricate a 1 cm^2^ square electrode.

The electrode was immersed in a 1 M solution of LiOH for 24 h prior to conducting electrochemical performance tests. Electrochemical performance testing was executed using a three-electrode system. This system included a Hg/HgO electrode as the reference electrode, a 1 cm × 1 cm piece of Pt metal as the counter electrode, and a CHI660E electrochemical workstation to perform CV, GCD, and EIS. EIS measurements were conducted at the open-circuit voltage over a frequency range from 1∼10^5^ Hz.

## 3 Results and discussion

### 3.1 The effect of heat treatment on the pore structure of trace PR

#### 3.1.1 Phase analysis

**FIGURE 1:**
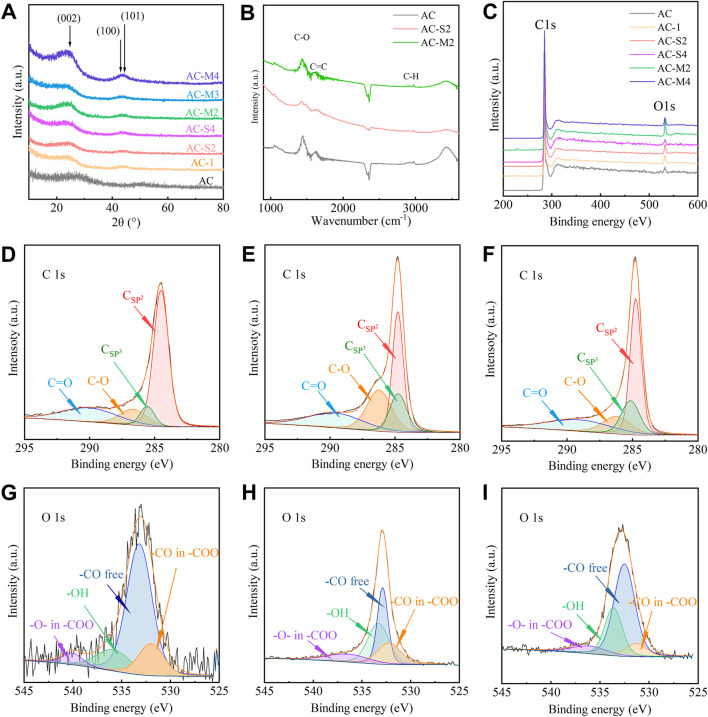
Performance characterization of carbon samples: **(A)** XRD curves of AC and samples under heat treatment; **(B)** FTIR of AC, AC−S2, and AC−M2; **(C)** XPS curves of AC and samples under heat treatment; **(D–F)** C1s curves of AC, AC−S2, and AC−M2; **(G–I)** O1s curves of AC, AC−S2, and AC−M2.

The structural composition of the coal-derived PC was analysed using X-ray diffraction (XRD), with the results presented in Figure 1A. As can be seen from the figure, after the PR heat treatment, the characteristic peak shifts to the left, indicating an increase in the interlayer spacing corresponding to the (002) crystal plane. Additionally, the figure also shows that the width of this characteristic peak narrows after the PR heat treatment, suggesting that the interlayer spacing of carbon microcrystals in the carbon material becomes more uniform. This indicates the formation of more regular carbon microcrystals, resulting in a decrease in the degree of amorphicity. All samples exhibited a broad diffraction peak around 26°, corresponding to the (002) plane of graphite. Based on Bragg’s law (Khani and Moradi, 2013), the interlayer spacing (d_002_) of the carbon layers in the samples was calculated, as shown in Table 2. The d_002_ of the activated carbon (AC) was 0.355 nm, whereas the d_002_ of the heat-treated samples was higher. For the samples subjected to a single heat treatment, d_002_ decreased with increasing PR content, indicating a reduction in interlayer spacing. Compared to the d_002_ of AC, the d_002_ of the heat-treated samples remained higher. For the samples undergoing multiple heat treatments (AC-M2, AC-M3, and AC-M4), the d_002_ was approximately 0.369 nm, suggesting that multiple heat treatments did not significantly alter the carbon interlayer spacing.

**TABLE 2:** Compositional structure of coal-derived PC samples.

Sample	d_002_ nm	SSA_BET_ (m^2^·g^-1^)	SSA_DFT_ (m^2^·g^-1^)	SSA_<0.65_ (m^2^·g^-1^)	SSA_0.65∼2_ (m^2^·g^-1^)	SSA_2∼5.5_ (m^2^·g^-1^)	V_DFT_ (cm^3^·g^-1^)	C (%)	O (%)
AC	0.355	1089	1198	553	388	375	0.67	97.10	2.90
AC-1	0.389	1485	1528	348	472	443	0.83	95.42	4.58
AC-S2	0.387	1501	1286	361	560	478	0.87	95.83	4.17
AC-S4	0.372	1501	1479	382	525	483	0.92	97.17	2.83
AC-M2	0.369	1497	1453	353	565	504	0.89	94.25	5.75
AC-M3	0.368	1482	1451	160	561	577	0.82	95.75	4.25
AC-M4	0.368	1418	1446	509	567	348	0.80	95.26	4.74

#### 3.1.2 Surface functional group analysis

To investigate the surface functional groups of the samples, Fourier transform infrared spectroscopy (FTIR) was employed, and the spectra of AC, AC-S2, and AC-M2 are shown in Figure 1B. Three characteristic peaks were observed at 2,930 cm^−1^, 1630 cm^−1^, and 1450 cm^−1^, corresponding to the symmetric stretching vibrations of C-H, C-O, and C=C, respectively, indicating that carbon is the primary component of the material (Ţucureanu et al., 2016). This suggests the presence of graphitic carbon atoms and the possible existence of oxygen-containing functional groups on the surface.

To further confirm the types of surface functional groups, X-ray photoelectron spectroscopy (XPS) analysis was conducted on AC and the heat-treated samples, as shown in Figure 1C. Characteristic peaks were observed at binding energies of 284.1 eV and 532 eV, corresponding to the C1s and O1s peaks, respectively (Levi et al., 2015; Paredes et al., 2021). The surface elemental compositions of C and O are summarised in Table 2.

The XPS spectra in Figure 1C revealed that for the singly heat-treated samples, AC-S2 exhibited the highest oxygen content at 95.83%, surpassing that of AC. This finding indicates that with increasing PR content, the oxygen content first increased and then decreased. For the multiple heat-treated samples, the carbon content remained similar. Notably, despite having the same total PR content, the carbon content of AC-S4 (97.17%) was higher than that of AC-M4 (95.26%), suggesting that with a fixed precursor amount, multi-step thermal treatments led to a reduction in carbon content.

Further analysis of surface functional groups was performed on AC-S2, the sample with the highest oxygen content among the single-step thermal treatment samples. The C1s spectrum in Figure 1E identified four distinct carbon states (Levi et al., 2015; Moreno-Castilla et al., 2000): a peak at 284.5 eV corresponding to sp2 graphitic carbon, peaks between 285.5 and 286 eV representing sp3 carbon, peaks between 287 and 289 eV indicating C-O bonds, and a peak at 290 eV attributed to C=O groups. These carbon species were consistent with those observed in the C1s spectrum of AC shown in Figure 1D. The O1s spectrum in Figure 1H confirmed the presence of various oxygen species on the coal-derived PC surface: peaks at 531.0–531.9 eV related to free -CO groups, peaks at 532.3–532.8 eV corresponding to -CO in ester or carboxyl groups, peaks at 533.1–533.8 eV associated with -O- groups in ester or carboxyl structures, and peaks at 530.5–536.5 eV attributed to -OH groups (Levi et al., 2015; Moreno-Castilla et al., 2000). These results demonstrate that the heat-treated carbon samples contained -OH, -CO, and -COO groups, consistent with the oxygen species identified in AC’s O1s spectrum shown in Figure 1G.

Surface functional group analysis was also performed on AC-M2, the sample with the highest oxygen content among the multi-step thermal treatments samples. The C1s spectrum in Figure 1F confirmed the presence of four carbon states (Levi et al., 2015; Moreno-Castilla et al., 2000), similar to those in AC, including sp2 carbon, sp3 carbon, C-O and C=O bonds. The O1s spectrum in Figure 1I verified the existence of various oxygen species, mirroring the oxygen functionalities observed in AC-S2. Both FTIR and XPS results indicate the presence of -OH, -CO, and -COO groups on the surface of PC. These findings suggest that the types of functional groups present on activated carbon surfaces remained unaffected by either single-step or multi-step heat treatments.

#### 3.1.3 Pore structure analysis

**FIGURE 2:**
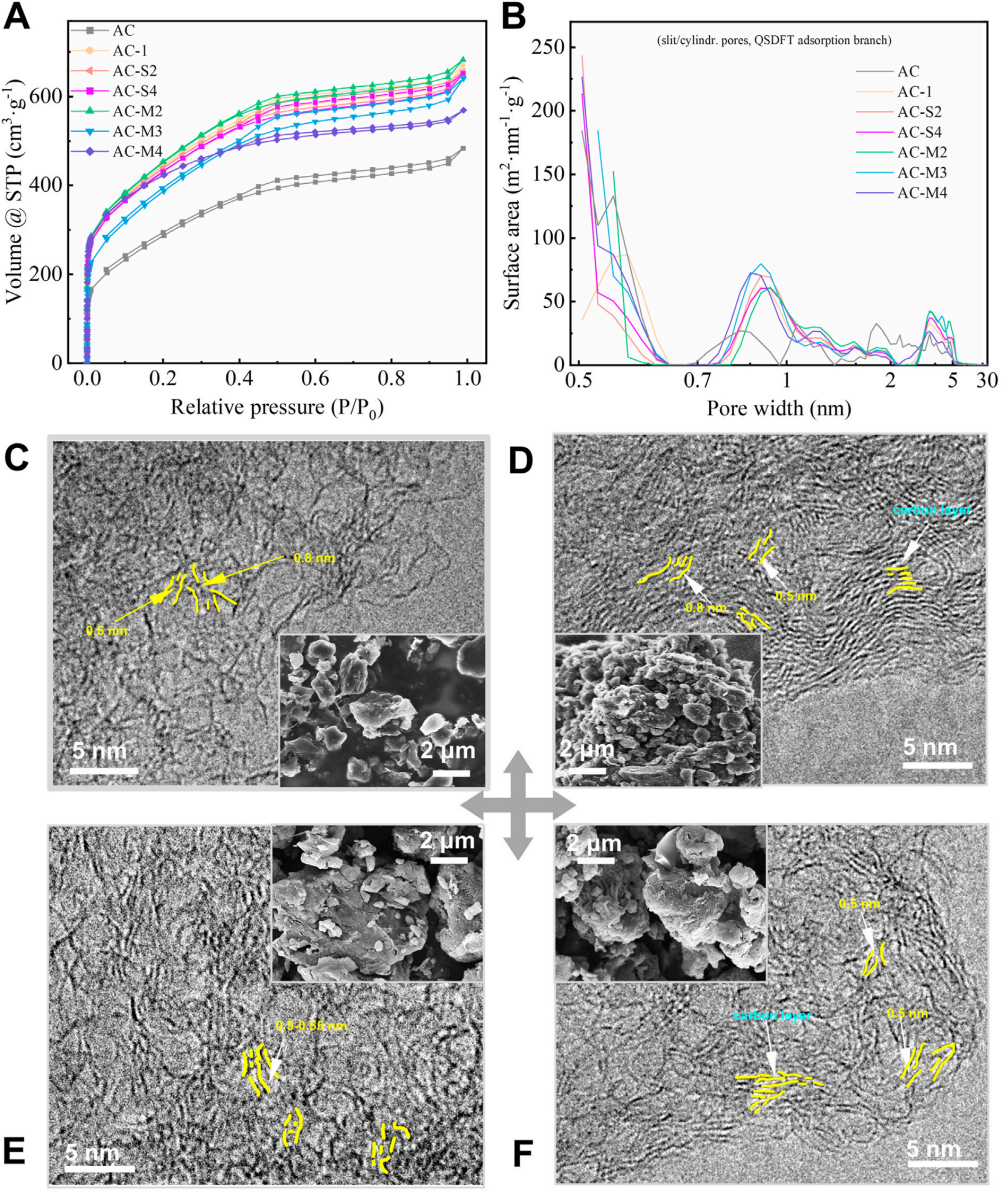
Pores distribution and microstructure evolution characterization of samples: **(A)** Isothermal adsorption curve of nitrogen at 77K; **(B)** The pore structure distribution curve of the samples; **(C–F)** microstructure image of AC, AC-S4, AC-M2 and AC-M4.

Nitrogen adsorption isotherms at 77 K were conducted on AC and the heat-treated samples, with the results shown in Figure 2A. All adsorption curves were consistent with type IV A isotherms, indicating the presence of both micropores and mesopores (Thommes et al., 2015). Notably, a hysteresis loop was observed within the P/P_0_ range of 0.35–0.95, characterised by a symmetric H4-type loop, suggesting the presence of slit-shaped mesopores (Thommes et al., 2015). Compared to AC, the heat-treated samples exhibited increased adsorption volumes, implying that carbon heat treatment enhanced the overall pore structure.

Further analysis of the nitrogen adsorption isotherms using the slit/cylindrical pores@QSDFT model, which provided the highest accuracy, yielded the pore size distribution curves shown in Figure 2B. The pore distribution of AC was concentrated within the ranges of 0.5–0.65 nm, 0.65–0.95 nm, 0.95–2 nm, and 2–5.5 nm. In contrast, the heat-treated samples exhibited a more focused pore distribution, with an increase in transitional pores in the range of 2.5–5 nm and a rise in micropores within 0.50–0.56 nm and 0.81–1.0 nm, while the pore volume in the 1.5–2 nm range decreased. Among these changes, the most significant effect of heat treatment was observed in the micropores smaller than 1 nm, warranting further investigation.

#### 3.1.4 Microscopic morphology analysis

To further evaluate the morphology of the coal-derived PC samples, the microstructures of AC, AC-S4, AC-M2, and AC-M4 were examined using scanning electron microscopy (SEM) and transmission electron microscopy (TEM), as shown in Figures 2C–F, respectively. The SEM images reveal that the samples exhibit a granular powder morphology. For single-step thermal treatment, samples prepared with a higher amount of PR exhibit significantly smaller particle diameters, as shown in Figure 2D. In the case of multiple thermal treatments, the particle size in Figure 2F is smaller than that in Figure 2E as the number of treatment cycles increases. These observations consistently indicate that a higher amount of PR leads to a reduction in particle diameter. This observation can be attributed to the gases generated during the pyrolysis of a substantial amount of PR, which disrupt the carbon particle structure.

TEM imaging demonstrates that the carbon samples, both before and after PR heat treatment, consist of few-layer graphene sheets. The internal pores are primarily slit-like gaps between the basal planes of these graphene sheets. For instance, in the AC-S4 sample, which underwent a single heat treatment with a larger quantity of PR, the graphene sheets are arranged in parallel, forming slit like gaps of approximately 0.5 nm between the basal planes (Figure 2D). In contrast, for samples subjected to multiple heat treatments, it is evident that as the number of heat treatment steps increases, the graphene sheets become smaller, and fewer stacked layers are observed, as shown in Figures 2E,F. These sheets exhibit varying degrees of curvature. Notably, as the number of heat treatment steps increases, the population of 0.5 nm pores also rises. For example, the area of <0.5 nm pores in AC-S4 is 213 m^2^⋅g^-1^, whereas in AC-M4 obtained after multiple heat treatments it increases to 227 m^2^⋅g^-1^ (Figure 2B).

In summary, by introducing extremely low levels (0.01%–1% by mass of activated carbon) of PR, we can enhance the porosity of carbon materials in the range of 0.5–0.55 nm and 2–5.5 nm during activation, without significantly increasing the content of oxygen functional groups. This allows us to satisfy the research requirements for pore size and lithium-ion capacitance performance.

### 3.2 Periodic pore distribution induced by thermal treatment

#### 3.2.1 Periodic distribution of pores

**FIGURE 3:**
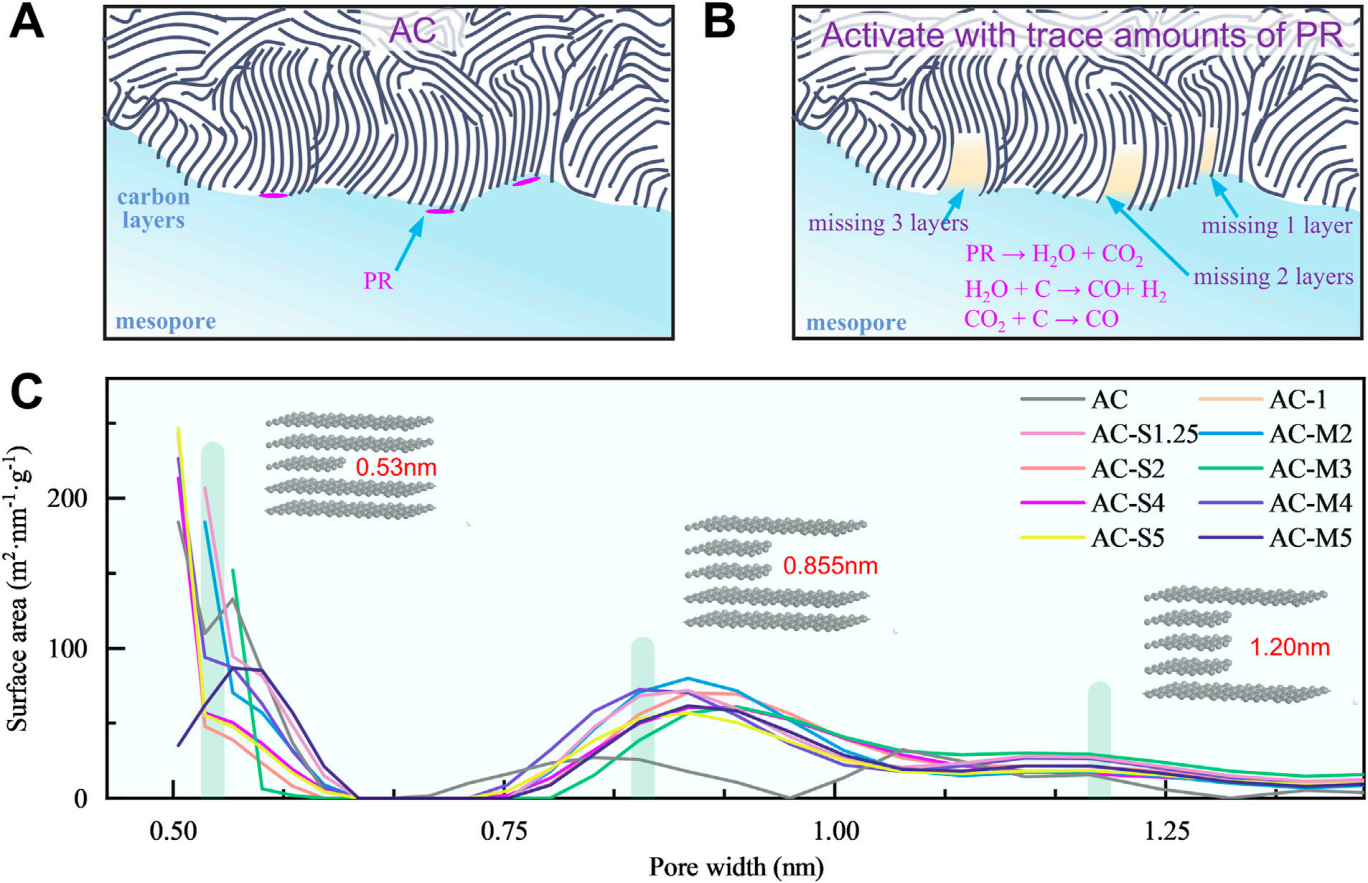
Periodic pore distribution phenomenon in PC: **(A)** The pore structure of AC; **(B)** Mechanism of PR activation heat treatment for removing graphite layer and forming pore structure; **(C)** Periodic distribution pattern of pore structure in samples.

The pore structure analysis of coal-derived PCs, as shown in Figure 2B, reveals that the distribution of carbon micropores is not random but rather concentrated around 0.53 nm, 0.855 nm, and 1.20 nm. Further investigation, incorporating the TEM images of the samples in Figure 2C–F, confirms that the PCs consist of carbon microcrystals composed of several layers of graphene-like structures, as illustrated in the model in Figure 3A. The atomic thickness of the carbon layers is approximately 0.14 nm (corresponding to the carbon atomic diameter), while the interlayer spacing of graphite is 0.335 nm (Zhang et al., 2024). When a carbon microcrystal formed by three layers of graphene undergoes the removal of the middle layer, the internal spacing expands to 0.53 nm-a process described as the delamination activation mechanism, as shown in Figure 3B. Similarly, the absence of two or three layers results in the formation of pores around 0.855 nm and 1.20 nm, respectively. This periodic distribution trend of pores is depicted in Figure 3C. It is worth noting that the carbon layers in PC are not perfectly parallel graphene sheets. As a result, the pore sizes obtained after delamination may slightly deviate from the theoretical values, leading to pores that are either larger or smaller than the predicted dimensions. For pores larger than 1.5 nm, as shown in Figure 2B, the specific surface area in the 2.5-5 nm range increases after thermal treatment, indicating that the heating of phenol-formaldehyde resin promotes the formation of larger mesopores.

Correspondingly, we compiled the pore size distribution curves of caol-derived PCs reported in relevant literature. The specific micropore distributions are summarised in Table 3, which reveals a similar periodic pattern in pore distribution (see Supplementary Material S1l for pore distributions of the samples reported in the literature) (Lee et al., 2015; Li et al., 2016; Shi et al., 2017; Su et al., 2018; Wang et al., 2020; Xia et al., 2025; Yang et al., 2022; Zhuang et al., 2021). As shown in Table 3, the pore size distribution of these coal-derived PCs exhibits a distinct concentration trend. For instance, in the sample “450” prepared by Li et al., (2016), the pores are predominantly distributed at 0.56 nm, 0.82 nm, and 1.15 nm, aligning with the pore sizes formed by the removal of 1, 2, and three carbon layers, respectively. Across all activated PCs listed, the formation of periodic micropores consistently correlates with the number of missing carbon layers.

**TABLE 3:** Compositional structure of coal-derived PC samples.

Samples	Raw material	Preparation method	Pore size {nm}			Ref
			Remov 1 layer	Remov 2 layers	Remov 3 layers	
CC	lignite	acidation	0.63	0.85	-	Li et al. (2016)
450		Acid + ZnCl_2_ activation	0.56	0.82	1.15	Li et al. (2016)
MSP-20	coal tar pitch	KOH activation	0.6	-	-	Lee et al. (2015)
CTP-93		KOH activation	0.65	-	1.05	Lee et al. (2015)
CTP-PP61	coal tar pitch + petroleum tar pitch	KOH activation + carbon deposition	0.65	-	1.12	Lee et al. (2015)
CTP-PP41		KOH activation + carbon deposition	0.65	-	1.1	Lee et al. (2015)
CTP-PP11		KOH activation + carbon deposition	0.65	-	1.1	Lee et al. (2015)
PC-3	coal tar pitch	KMnO_4_ oxidation	-	-	1.1	Zhuang et al. (2021)
3DPC-2		KMnO_4_ oxidation + NaCl template	-	-	1.1	Zhuang et al. (2021)
3DPC-3		KMnO_4_ oxidation + NaCl template	-	0.85	1.1	Zhuang et al. (2021)
3DPC-4		KMnO_4_ oxidation + NaCl template	-	0.85	1.1	Zhuang et al. (2021)
HPC-CCP	coal tar pitch	Heating after mixing with CCP	0.62	0.85	1.1	Zhuang et al. (2021)
HPC-CCP/PAA		Heating after mixing with CCP + PAA		0.84	-	Wang et al. (2020)
CS1	resorcinol-formaldehyde resin	Carbonization, CO_2_ activation	0.53	0.85	1.21	Shi et al. (2017)
CS2		Carbonization, CO_2_ activation	0.53	0.85	1.23	Shi et al. (2017)
CK1	Poly (sodium 4-styrenesulfo nate)	KOH activation	0.56	0.87	1.23	Shi et al. (2017)
CK3		KOH activation		0.85	1.15	Su et al., 2018
CTS-NaNH_2_(1:1)-600	chitosan	NaNH_2_		0.85	1.23	Yang et al. (2022)
CTS-NaNH_2_(2:1)-900		NaNH_2_		0.86		Xia et al. (2025)

Compared with traditional activation methods using KOH, CO_2_, and similar agents, the use of ultra-low concentrations of PR enables more precise control over pore structure. This allows the pore architecture to be tailored to meet the requirements of various application scenarios, thereby enhancing the overall performance of PCs. This PR-based pore regulation strategy holds great promise for the development of PC materials.

#### 3.2.2 Mechanism of periodic pore distribution formation

We further analysed the delamination phenomenon occurring during the heat treatment process of PR. It was found that both the deposition amount and the number of depositions affected the pore structure. This influence is manifested in the transition pores and micropores in the following ways:

1. Activation Leads to Concentrated Distribution of Transition Pores
  During heat treatment, PR undergoes pyrolysis, generating H_2_O and CO_2_. These gases react with the pore walls, producing CO and H_2_, which disrupt the pore walls and further promote pore development. The increase in transition pores in the range of approximately 2.5–5.5 nm, as shown in Figure 2B, reflects this trend.
2. Delamination Activation Forms Periodic Micropores
  We further analysed the delamination phenomenon that occurred during the experimental process. During heat treatment, PR produces gases such as CO_2_ and H_2_O, which react with the carbon layers, activating and etching the graphite layers within the carbon microcrystals. By selectively removing different numbers of graphite layers, periodic pore distributions are formed. This process, illustrated in Figure 3B, demonstrates that the delamination activation induced by a small amount of PR is crucial to pore formation.

Specifically, for example, in the case of AC-1, as PR was added, 1–3 layers of heat-treated carbon in the micropores were selectively etched, resulting in pore sizes of 0.535 nm, 0.86 nm, and 1.19 nm, respectively. For the single-step, PR addition increased, such as in AC-S4, the CO_2_ and H_2_O gases generated during the process led to enhanced activation, allowing the etching of 1–2 graphite layers in the micropores more easily, thereby increasing micropores with sizes of 0.53 nm and 0.855 nm. With multi-step, as in AC-M4, the continuous generation of CO_2_ and H_2_O gases from repeated heat treatments resulted in ongoing etching of carbon layers. This not only increased the micropores with sizes of 0.53 nm and 0.853 nm but also caused damage to the carbon layers, leading to shorter carbon sheets. For both AC-M4 and AC-S4, the total amount of PR introduced was 0.008 g; however, as shown in Figure 2F, the carbon sheet length in AC-M4 was shorter than that in AC-S4, as seen in Figure 2D. This difference arises from the multiple heat treatment steps that caused delamination and pore formation, which also damaged the graphite layers, as illustrated in Figure 2E.

In conclusion, it can be seen that when a small amount of PR, ranging from 0.01% to 1% of the mass of activated carbon, is introduced, heat treatment can adjust the pore structure by increasing the number of micropores. By varying the carbon precursor mass and the number of heat treatment steps, nanoscale pore control can be achieved to meet the requirements of various applications for PC materials.

### 3.3 Capacitive performance of porous carbon

**FIGURE 4:**
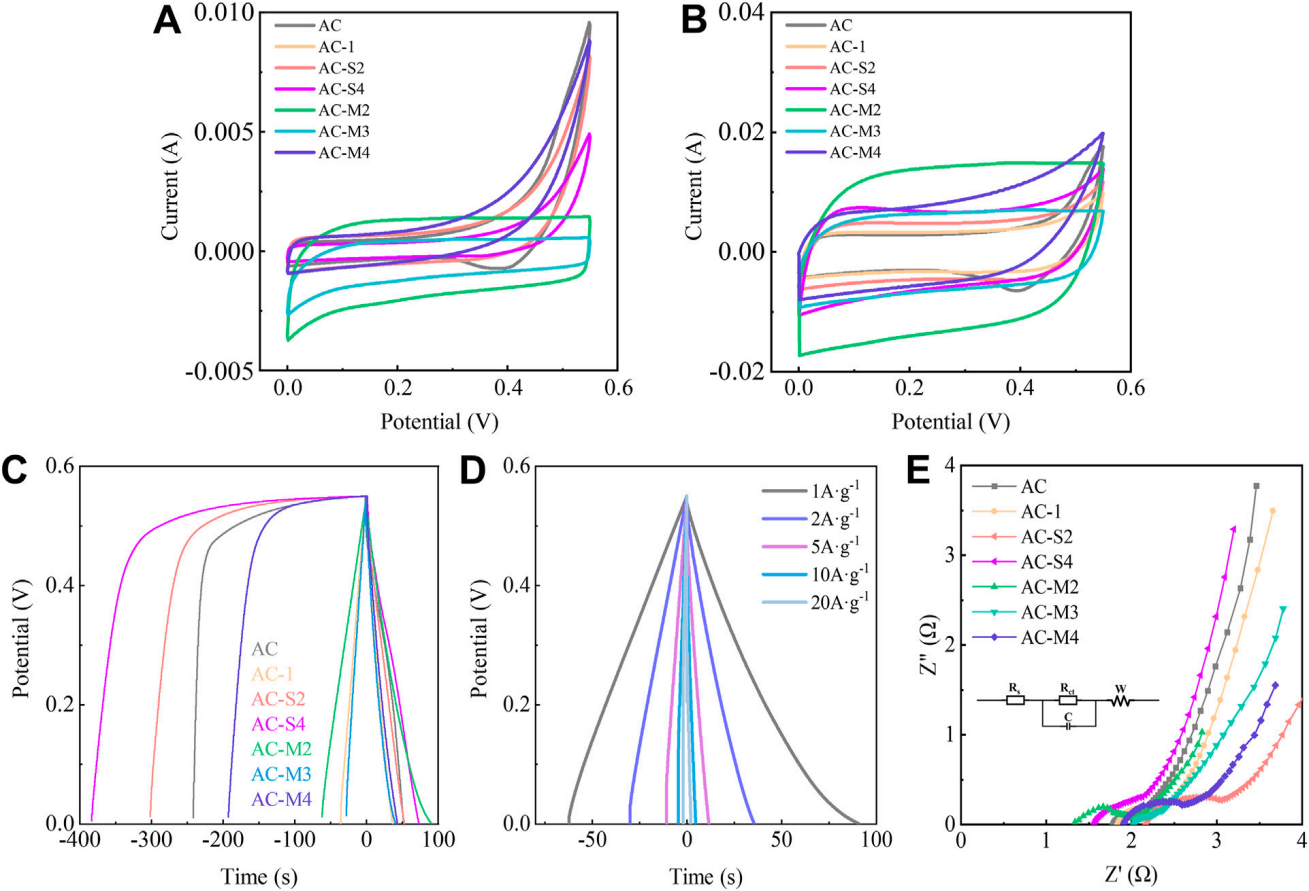
Capacitive performance of porous carbon samples: **(A)** and **(B)** CV curves of samples at 5  mV s^-1^ and 50  mV s^-1^; **(C)** GCD curves of samples at 1 A·g^-1^; **(D)** GCD curves of AC-M2 at different current densities; **(E)** The Nyquist curve of the samples measured by EIS.

To determine the optimal pore size for LICs, capacitive performance of coal-derived PCs was evaluated using 1 M LiOH as the electrolyte in a three-electrode configuration, as shown in Figure 4.

#### 3.3.1 Cyclic voltammetry analysis (CV) analysis

The CV results of the coal-derived PCs at a scan rate of 5 mV⋅s^-1^ are shown in Figure 4A. The AC sample exhibited a reduction peak at 0.45–0.55 V and an oxidation peak at 0.33–0.42 V, indicating reversible pseudo capacitance. After heat treatment, the CV curves of the samples adopted a nearly rectangular shape, signifying the formation of a well-defined electric double-layer structure. The CV results at 50 mV⋅s^-1^, shown in Figure 4B, reveal the symmetry of the CV curves improved with increasing scan rates, suggesting enhanced electric double-layer formation (Mattsson et al., 2004). Among the samples, AC-M2 displayed the most symmetric CV curve, highlighting its superior Li-ion double layer energy storage capability.

#### 3.3.2 Galvanostatic charge-discharge (GCD) analysis

GCD tests were performed to evaluate the specific capacitance of coal-derived PCs at various current densities. The capacitances are summarised in Table 4, with the GCD curves at 1 A⋅g^-1^ shown in Figure 4C. At 1 A⋅g^-1^, the pre-heat-treated AC sample exhibited a capacitance of 100 F⋅g^-1^. The sample with the highest single PR addition, AC-S4, showed a significantly reduced capacitance of 67 F⋅g^-1^, whereas AC-1, with the smallest PR addition, achieved a higher specific capacitance of 104 F⋅g^-1^. Among the samples subjected to multiple heat treatments, AC-M2 (after two-steps heat treatments) exhibited the highest capacitance, reaching 163.6 F⋅g^-1^, surpassing the reported capacitance of coal-derived carbon materials in the literature (Supplementary Material S2l) (Bober et al., 2017; Karamanova et al., 2020; Krause et al., 2011; Shaibani et al., 2017; Stepniak and Ciszewski, 2010). AC-M2 maintained a capacitance retention of 90.21% after 500 cycles, as evidenced by the GCD curves in Supplementary Material S3l. As representative commercial carbon electrode materials for supercapacitors, YP-50F and YP-80F produced by Kuraray exhibit specific capacitances of only 113 F⋅g^-1^ and 114 F⋅g^-1^, respectively, at a current density of 0.9 A⋅g^-1^. In comparison, the AC-M2 material synthesized in this work demonstrates superior capacitive performance (Karamanova et al., 2020).

**TABLE 4:** Electrochemical performance of coal-derived PC samples.

Sample	C_1A_ (F·g^-1^)	C_20A_/C_1A_ (%)	Diffusion coefficients (10^−16^)	Rs (Ω)	Rct (Ω)
AC	100	10.72	8.46	1.73	0.46
AC-1	104	34.03	5.67	1.76	0.54
AC-S2	63	21.75	3.48	1.82	0.96
AC-S4	67	37.43	2.89	1.52	0.62
AC-M2	164	45.29	18.54	1.35	0.77
AC-M3	73	24.86	5.33	1.93	0.45
AC-M4	84	36.01	9.72	1.79	0.95

Further analysis of AC-M2, the sample with the highest capacitance, was conducted. Figure 4D displays its GCD curves at different current densities, revealing a symmetrical triangular shape consistent with the symmetry observed in the CV curves. This confirms that the capacitance of AC-M2 primarily stems from electric double-layer capacitance.

#### 3.3.3 Electrochemical impedance spectroscopy (EIS) analysis

EIS analysis was performed on the coal-derived PCs, and the resulting Nyquist plots are shown in Figure 4E. The curves comprise a semicircle in the high-frequency region and a straight line in the low-frequency region. In the high-frequency region, the left intercept of the semicircle corresponds to the electrode’s surface resistance (Rs), while the semicircle’s diameter represents the charge transfer resistance (Rct) (Chen et al., 2009). The calculated values are listed in Table 3. A larger Rs​ indicates poorer conductivity of the electrolyte solution and greater resistance to ion migration, while a larger Rct reflects a more difficult charge transfer process and a slower electrode reaction rate. Post heat treatment, the Rct values of the samples were relatively similar; however, AC-M2 exhibited the smallest Rs at 1.35 Ω, indicating the lowest internal resistance.

In the low-frequency region, a straighter line approaching a 45° angle suggests better ion diffusion. The diffusion coefficient was derived from the Nyquist plots (Chen et al., 2009), with the calculation details provided in Supplementary Material S4l. As shown in Table 4, AC-S4 had the lowest diffusion coefficient at 2.89 × 10^−16^, likely due to the abundance of 0.5 nm pores. Conversely, samples with fewer 0.50 nm pores exhibited relatively higher diffusion coefficients, with AC-M2 achieving the highest diffusion coefficient at 18.54 × 10^−16^. These results suggest that 0.54 nm pores may be more favourable for ion diffusion, contributing to the improved capacitive performance.

### 3.4 Influence of pore structure on capacitive performance

The electrode materials of PC capacitors primarily rely on the formation of an electric double layer between the pore surfaces and electrolyte ions for energy storage. Therefore, by analysing the correlation between specific surface area, capacitance, and diffusion coefficient, the influence of pore size on Li-ion capacitor performance can be evaluated. Specifically, we employ the Pearson correlation coefficient (Cohen et al., 2009) to quantify the linear correlation between two variables. The coefficient is calculated using Equation 1:

$$r=\frac{\sum \left(x_{i}-\bar{x}\right)\left(y_{i}-\bar{y}\right)}{\sqrt{\sum {\left(x_{i}-\bar{x}\right)}^{2}\sum {\left(y_{i}-\bar{y}\right)}^{2}}}$$ (1)

**FIGURE 5:**
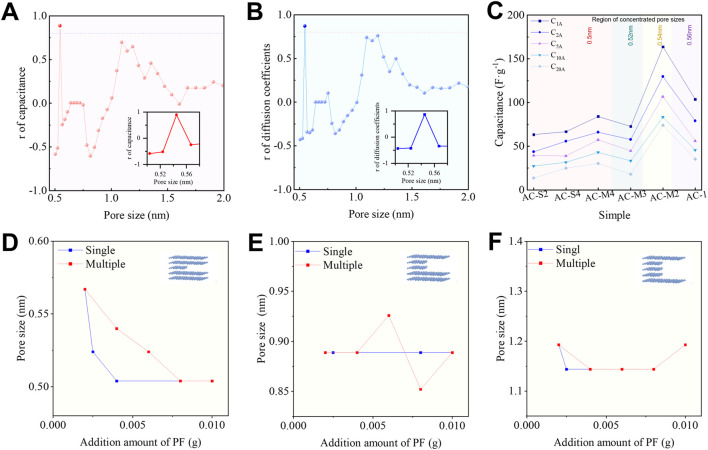
Influence of pore size on capacitive performance: **(A)** Correlation between pore size and electrochemical performance calculated using the Pearson correlation coefficient; **(B)** Correlation between pore size and electrochemical performance analyzed by distance correlation; **(C)** Rate performance of PCs at different current densities; **(D–F)** Effect of the PR mass ratio and the number of activation steps on the pore size resulting from the removal of intermediate one to three graphitic layers.

A Pearson correlation coefficient (r > 0.80) indicates a strong positive correlation between the independent and dependent variables. We analysed the correlation between the surface area of pores of different sizes and the specific capacitance, C_1A_, as shown in Figure 5A. The results reveal that when the pore size approaches 0.54 nm, the correlation between surface area and C_1A_ is strongest, with r = 0.8878, suggesting a highly significant relationship. The curve depicting the relationship between the 0.54 nm pore surface area and sample capacitance is provided in Supplementary Material S5A confirming, confirming that a larger 0.54 nm surface area corresponds to higher capacitance. Subsequently, we examined the correlation between pore surface area and diffusion coefficient, as shown in Figure 5A. The results demonstrate that the correlation is strongest when the pore size is around 0.54 nm, with r = 0.8706, indicating a robust positive relationship. The curve depicting the relationship between the 0.54 nm surface area and diffusion coefficient is shown in Supplementary Material S5B.

$$dCor\left(X,Y\right)=\frac{\text{dCov}\left(X,Y\right)}{\sqrt{\text{dCov}\left(X,X\right)\cdot \text{dCov}\left(Y,Y\right)}}$$ (2)

To obtain a more comprehensive and accurate correlation analysis, relying solely on linear correlation (Pearson correlation coefficient) may be limited. Therefore, distance correlation was further introduced as a complementary method (Wang et al., 2015), as detailed in Equation 2. Unlike the Pearson coefficient, which can only identify linear relationships, distance correlation is capable of detecting any form of statistical dependence. The results of the distance correlation analysis are shown in Figure 5B, where it can be observed that when the pore size approaches approximately 0.54 nm, the correlations between surface area and both capacitance and diffusion coefficient are the strongest. This suggests that the larger the 0.54 nm pore surface area, the better the power performance of the sample. The best capacitive performance was observed when the 0.54 nm pore area increased.

Based on this, we correlated the capacitive performance measured at different current densities with the distribution of pore sizes, as shown in Figure 5C. It is evident that the AC-M2 sample, with a dominant pore size of 0.54 nm, exhibited the highest capacitance at 1 A⋅g^-1^, reaching 164 F⋅g^-1^. Furthermore, the capacitance retention at 20 A⋅g^-1^ was the highest at 45.29%, highlighting its superior power performance. Collectively, these findings suggest that the 0.54 nm pores—formed by the removal of a single graphite layer—are most conducive to enhancing Li-ion capacitive performance.

We further explored the relationship between PR content, activation method, and the pore sizes formed by the removal of 1–3 graphite layers, as shown in Figures 5D–F. It was observed that trace amounts of PR primarily influenced the formation of 0.50–0.56 nm pores (corresponding to the removal of a single graphite layer) while having a relatively minor effect on the pores formed by the removal of two or three layers. The total PR content was found to control the size of the single-layer pores: during single-step heat treatment, a PR mass ratio of 0.4% resulted in the formation of 0.50 nm pores, the smallest pore size measurable by the QSDFT model used in this study, as N_2_ adsorption cannot effectively detect pores smaller than 0.50 nm.

In the case of multiple heat treatments, the rate of pore size reduction slowed. The minimum 0.50 nm pores were only achieved when the PR mass ratio reached 0.8%. Therefore, the use of trace amounts of PR combined with multiple heat treatments provides a more effective approach to fine-tuning carbon micropores within the 0.50–0.56 nm range at the sub-nanometre scale. This method offers a novel strategy for fabricating Li-ion capacitor electrodes enriched with optimally sized pores, paving the way for the development of high-performance carbon-based materials for Li-ion storage.

## 4 Conclusion

This study investigates the regulation of pore structures in PC using trace amounts of PR resin through multiple heat treatments. The results show that the activation process during heat treatment removes internal graphitic layers, which constitutes the primary mechanism for micropore formation. The etching of one–three layers creates pores with diameters of approximately 0.53 nm, 0.85 nm, and 1.2 nm, resulting in a periodic distribution of micropores, a pattern commonly observed in PC materials. By analysing the relationship between carbon pore size and Li-ion capacitive performance, we identified 0.54 nm pores, formed by the removal of a single graphitic layer, as the optimal size for both Li-ion capacitance and diffusion coefficient. The coal-derived PC obtained through 2-steps heat treatments with PR contained a significant number of 0.54 nm pores, achieving a capacitance of 164 F⋅g^-1^, comparable to the highest levels reported in the literature. This study provides a theoretical basis for the precise control of pore structures in carbon electrode materials for LICs and contributes to the development of carbon materials with optimised pore architectures for improved electrochemical performance.

## Data Availability

The datasets presented in this study can be found in online repositories. The names of the repository/repositories and accession number(s) can be found in the article/Supplementary Material.
