# Supplementary material for: Trace phenol-formaldehyde resin activation mechanism of intermediate graphitic layer removal in carbon for enhanced Li-ion capacitor performance
Source: Front Chem. 2025 Sep 22;13:1592695. doi: 10.3389/fchem.2025.1592695 (PMC12498956; doi:10.3389/fchem.2025.1592695)
Supplement: Supplementary file 2 [file DataSheet1.docx]

Supplementary Material

**S1 Periodic pore distribution phenomenon in literature**


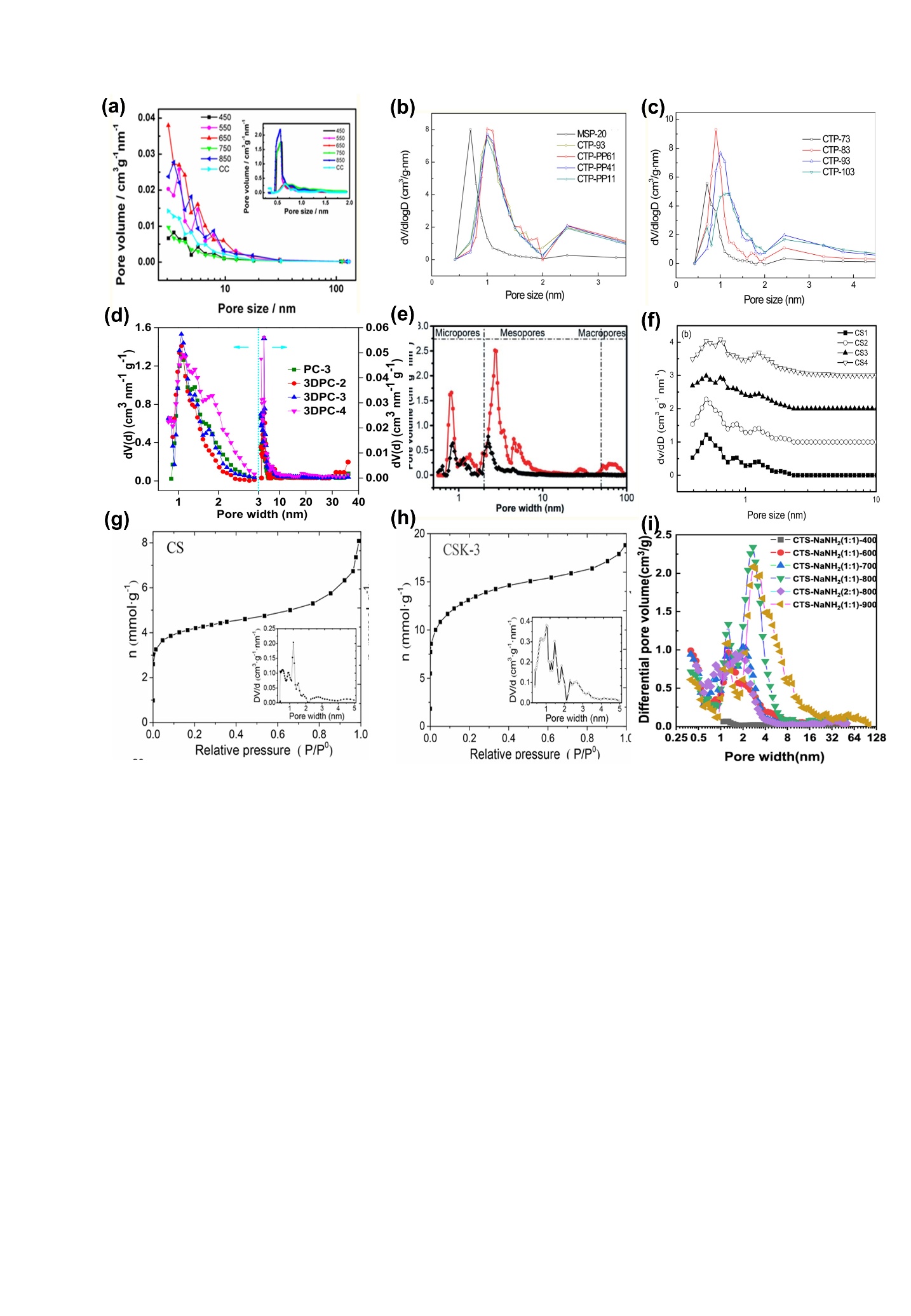


**Figure S1** Periodic pore distribution phenomenon in literature

(a): pore distribution of the sample in [1]; (b) and (c): pore distribution of the sample in [2]; (d): pore distribution of the sample in [3]; (e): pore distribution of the sample in [4]; (f): pore distribution of the sample in [5]; (g) and (h): pore distribution of the sample in [6]; (i): pore distribution of the sample in [7].

**S2** **Capacitive properties of porous carbon in this study versus references**

**Table S1** The capacitive performance of LIC carbon electrode material

| Raw materials | Samples | SSA_BET_  (m^2^·g^-1^) | Electrolyte solution | | Test methods | capacitance  (F·g^-1^) | | Reference |
| --- | --- | --- | --- | --- | --- | --- | --- | --- |
| bituminous coal | AC-M2 | 1497 | 1M LiOH | Three-electrode system | | | 164  (1A·g^-1^) | This work |
| coconut shell carbon | YP-50F  (Kuraray) | 1600 | 6M LiOH | Two-electrode system | | | 113  (0.9A·g^-1^) | [8] |
| anthracite | YP-80F  (Kuraray) | 2100 | 6M LiOH | Two-electrode system | | | 114  (0.9A·g^-1^) | [8] |
| PAF | MOLC | 1084 | LiOH | Two-electrode system | | | 35  (1A·g^-1^) | [9] |
| Polyaniline–phosphite | PANI-DPPH-C | <0.1 | 3M LiOH | Three-electrode system | | | 57.6  (5mV·s^−1^) | [10] |
| Activated carbon fiber | PP membrane | - | 4.6M LiOH | Three -electrode Swagelok-type cells | | | 108  (1A·g^-1^) | [11] |
| carbon black | SC3 | 1880 | 1M LiPF_6_ | Three -electrode Swagelok-type cells | | | 115  (5 mA·cm^−2^) | [12] |
| Natural graphite | MWCNTs | <1 | 2M LiOH | Three -electrode Swagelok-type cells | | | 40  (1A·g^-1^) | [13] |

**S3** **Cycle Performance of AC-M2**

**Figure S3** GCD curves of over 500 cycles

The GCD curves of sample AC-M2 at 1 A·g⁻¹ over 500 cycles are shown in **Figure S3**, demonstrating a capacitance retention of 90.21%.

**S4 Method for solving ion diffusion coefficient**

Analyzing the Nyqust curve (EIS). The diffusion coefficient is obtained using Eq. (1) and Eq. (2) [14].

$Z'={\sigma\omega}^{-0.5}$ (1)

$k=\frac{R^{2}T^{2}}{2A^{2}n^{4}F^{4}{C_{K}}^{2}\sigma^{2}}$ (2)

Where Z’ is Warburg impedance and σ is Warburg impedance factor. ω is angular-frequency. R represents the gas constant (R = 8.314 J·mol^-1^·K^-1^), T is the absolute temperature of the test environment, A is the area of the positive electrode material immersed in the electrolyte, n is the number of electrons transferred in the electrochemical reaction (n = 2), and F is the Faraday constant (F = 96500 C·mol).

**S5 The pore size most strongly correlated with capacitive performance**


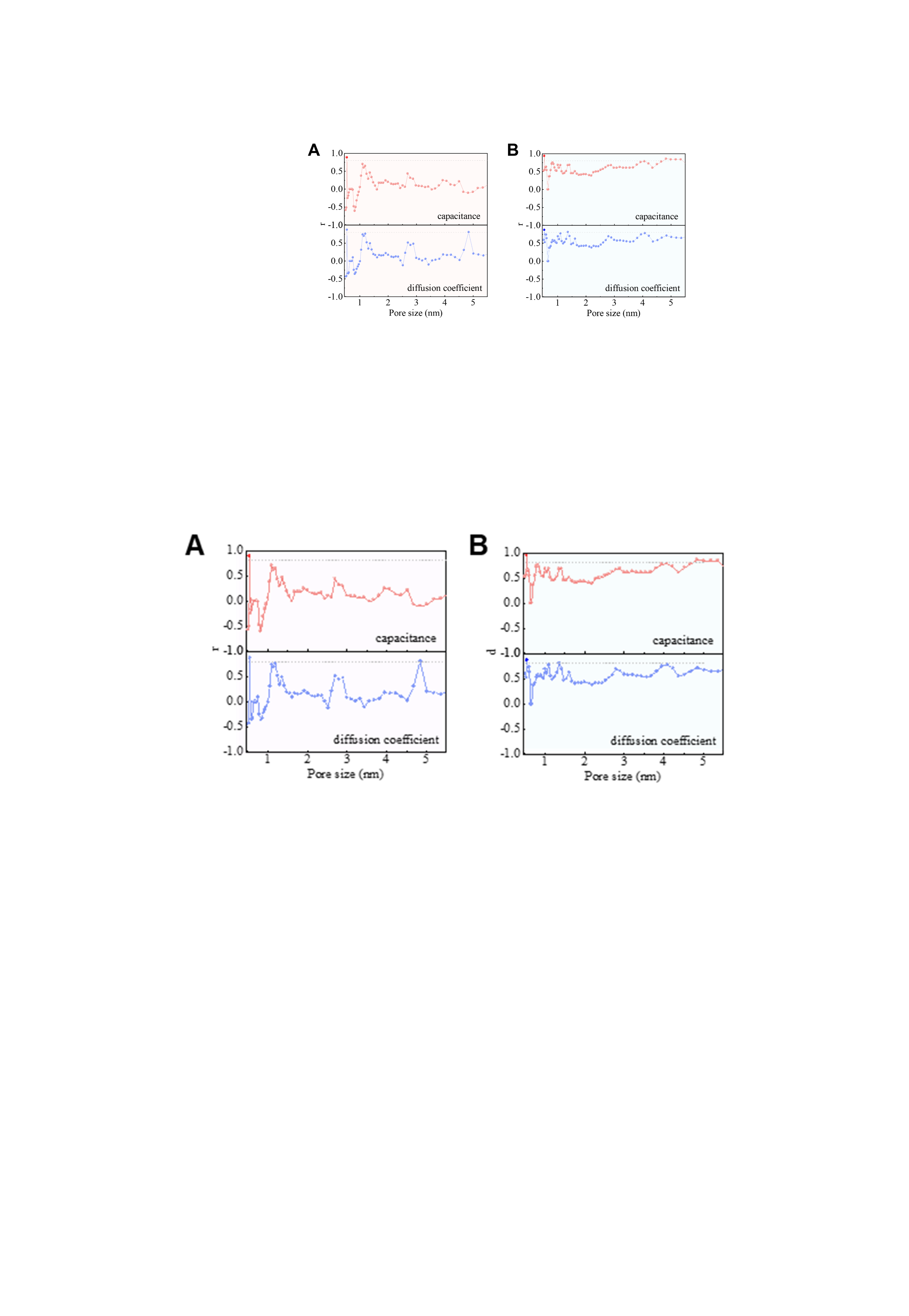


**Figure S5** Calculation results using Pearson coefficient (A) and distance analysis method (B)

**References**

1. Li L, Wang X, Wang S, Cao Z., Wu Z., Wang H., Gao Y., Liu J. Activated carbon prepared from lignite as supercapacitor electrode materials. *Electroanalysis* **2016***, 28(1*): 243-248.
2. Lee E., Kwon SH., Choi PR., Jung JC., Kim MS. Activated carbons prepared from mixtures of coal tar pitch and petroleum pitch and their electrochemical performance as electrode materials for electric double-layer capacitor. *Carbon Lett.* **2015***, 16(2)*: 78-85. **(Open Access)**
3. Zhuang QQ., Cao JP., Wu Y., Zhao XY., Wei YL., Yang ZH., Zhou Z., He ZM., Zhao YP., Bai HC. Direct synthesis of oxygen-enriched 3D porous carbons via NaCl template derived from oxidized coal tar pitch for excellent cycling stability electric double layer capacitor. *J. Power Sources* **2021***, 508*: 230330.
4. Wang H., Zhou C., Zhu H., Li Y., Wang S., Shen K. Hierarchical porous carbons from carboxylated coal-tar pitch functional poly (acrylic acid) hydrogel networks for supercapacitor electrodes. *RSC Adv.* **2020***, 10*(2): 1095-1103. **(Open Access)**
5. Shi J, Yan N, Cui H, et al. Sulfur doped microporous carbons for CO2 adsorption. *J. Environ. Chem. Eng*, **2017**, *5*(5): 4605-4611.
6. Su W, Yao L, Ran M, et al. Adsorption properties of N2, CH4, and CO2 on sulfur-doped microporous carbons. *Journal of Chemical & Engineering Data*, **2018**, *63*(8): 2914-2920.
7. Yang C, Zhao T, Pan H, et al. Facile preparation of N-doped porous carbon from chitosan and NaNH2 for CO2 adsorption and conversion. *Chem. Eng. J*., **2022**, *432*: 134347..
8. Karamanova B, Stoyanova A, Shipochka M, et al. Effect of alkaline-basic electrolytes on the capacitance performance of biomass-derived carbonaceous materials. *Materials*, **2020**, *13*(13): 2941.
9. Shaibani M, Smith S J D, Banerjee P C, et al. Framework-mediated synthesis of highly microporous onion-like carbon: energy enhancement in supercapacitors without compromising power. *J. Mater. Chem. A*, **2017**, *5*(6): 2519-2529.
10. Bober P, Trchová M, Morávková Z, et al. Phosphorus and nitrogen-containing carbons obtained by the carbonization of conducting polyaniline complex with phosphites. *Electrochim. Acta*, **2017**, *246*: 443-450.
11. Stepniak I, Ciszewski A. New design of electric double layer capacitors with aqueous LiOH electrolyte as alternative to capacitor with KOH solution. *J. Power Sources*, **2010**, *195*(9): 2564-2569.
12. Krause A, Kossyrev P, Oljaca M, et al. Electrochemical double layer capacitor and lithium-ion capacitor based on carbon black. *J. Power Sources*, **2011**, *196*(20): 8836-8842.
13. Kumar A, Kumar N, Sharma Y, et al. Synthesis of free-standing flexible rGO/MWCNT films for symmetric supercapacitor application. *Nanoscale Res. Lett.*, **2019**, *14*: 1-17.
14. Chen M, Xuan H, Zheng X, et al. N-doped mesoporous carbon by a hard-template strategy associated with chemical activation and its enhanced supercapacitance performance. *Electrochim. Acta*, **2017**, *238*: 269-277.
